# Sexual Phenotype Differences in *zic2* mRNA Abundance in the Preoptic Area of a Protogynous Teleost, *Thalassoma bifasciatum*

**DOI:** 10.1371/journal.pone.0023213

**Published:** 2011-08-03

**Authors:** Katherine McCaffrey, Mary Beth Hawkins, John Godwin

**Affiliations:** Department of Biology and W.M. Keck Center for Behavioral Biology, North Carolina State University, Raleigh, North Carolina, United States of America; National University of Singapore, Singapore

## Abstract

The highly conserved members of the *zic* family of zinc-finger transcription factors are primarily known for their roles in embryonic signaling pathways and regulation of cellular proliferation and differentiation. This study describes sexual phenotype differences in abundances of *zic2* mRNA in the preoptic area of the hypothalamus, a region strongly implicated in sexual behavior and function, in an adult teleost, *Thalassoma bifasciatum*. The bluehead wrasse (*Thalassoma bifasciatum*) is a valuable model for studying neuroendocrine processes because it displays two discrete male phenotypes, initial phase (IP) males and territorial, terminal phase (TP) males, and undergoes socially-controlled protogynous sex change. Previously generated microarray-based comparisons suggested that *zic2* was upregulated in the brains of terminal phase males relative to initial phase males. To further explore this difference, we cloned a 727 bp sequence for neural *zic2* from field-collected animals. Riboprobe-based *in situ* hybridization was employed to localize *zic2* signal in adult bluehead brains and assess the relative abundance of brain *zic2* mRNA across sexual phenotypes. We found *zic2* mRNA expression was extremely abundant in the granular cells of the cerebellum and widespread in other brain regions including in the thalamus, hypothalamus, habenula, torus semicircularis, torus longitudinalis, medial longitudinal fascicle and telencephalic areas. Quantitative autoradiography and phosphorimaging showed *zic2* mRNA hybridization signal in the preoptic area of the hypothalamus was significantly higher in terminal phase males relative to both initial phase males and females, and silver grain analysis confirmed this relationship between phenotypes. No significant difference in abundance was found in *zic2* signal across phenotypes in the habenula, a brain region not implicated in the control of sexual behavior, or cerebellum.

## Introduction

Zic family members encode zinc-finger transcription factors composed of five tandem Cys2His2 zinc-finger domains [1]. At least some teleost genomes encode seven *zic* genes, perhaps from a duplication event (zebrafish; *zic1, zic4, zic2a, zic5, zic3, zic6, and zic2b* structured in three pairs with one unpaired gene, respectively). However, *zic2* is one of the five highly conserved *zics* among vertebrate groups *(zic1-5)* [2]. These highly conserved *zic* family members play critical roles in upregulating proliferation factors and delaying differentiation during development [2] and in mammalian neurulation [3], [4]. Z*ic2* is one of the few genes, independent of the hedgehog pathway, linked to the severe and prevalent congenital malformation of the forebrain termed holoprosencephaly in humans (HPE) [2], [5]. Also related to development, retinal ganglion cell (RGC) expression of *zic2* patterns binocular vision by specifying the ipsilateral projection of RGCs [6]. Z*ic2* mRNA has been detected across a wide range of malignant tissues [7], [8], [9], [10], as well as found to be expressed in normal human brain and testicular tissue, but not kidney, skin, small intestine, pancreas, uterus, or lung [8]. Since most studies have focused on developmental roles for zic genes, we know relatively little about the role of these genes in mature tissues despite increasing evidence they play important roles in adults [11].

In the mature mammalian brain, *zic2* is strongly expressed in cerebellar granule cells in the hindbrain of rodent models and humans [4], [10], [12], [13], [14], [15], [16]. Some studies have described adult brain expression of *zic2* as restricted to the cerebellum and functioning as a marker for granule cell neurons and their precursors [15]. However, Aruga and collaborators [14] also detected weak *zic* mRNA signaling in the medial habenula nucleus, olfactory bulb, thalamus, and pontine nucleus of adult mice. Similarly, Brown and Brown [17] noted mouse *zic2* expression in the olfactory bulb, the rostral migratory stream (RMS), and the sub-ventricular zone (SVZ) in addition to hindbrain labeling.

Our objective in this study is to examine the abundance of neural *zic2* mRNA expression across the three sexual phenotypes of the bluehead wrasse, *Thalassoma bifasciatum*. The bluehead wrasse is a well-studied model for behavioral and neuroendocrine aspects of sex change and alternate sexual phenotypes. This species displays two discrete alternate male phenotypes, initial phase (IP) males and territorial, terminal phase (TP) males, and undergoes socially-induced sex change [18], [19], [20]. Terminal phase (TP) males are large bodied, brightly colored, and usually aggressively defend breeding sites where they court and spawn with females. Females and initial phase (IP) males are smaller in body size, are not aggressive, and share a similar yellow and brown coloration and are indistinguishable externally other than the dimorphic genital papilla. IP males obtain fertilizations through parasitic mating tactics (“sneak” or “streak” spawning) or by participating in group spawns [20]. Upon becoming socially dominant, both females and IP males become TP males [21], [22], [23]. The bluehead wrasse is therefore a model system that allows the study of neuroendocrinological processes underlying both rapid behavioral and gonadal sex change within a natural context.

Expression data from bluehead wrasse brain cDNA microarrays generated in our lab indicated that *zic2* was upregulated in terminal phase males relative to both females and IP males (Passador-Gurgel and Godwin, unpublished data). This study focuses on the preoptic area of the hypothalamus and the possibility that *zic2* mRNA abundance is elevated specifically in TP males relative to females and IP males in this key integrative area for male-typical courtship and sexual behavior [24], [25], [26]. A second goal was to characterize the distribution of *zic2* mRNA expression in the brain of the bluehead wrasse using *in situ* hybridization.

## Results

### Cloning of *zic2* cDNA

We amplified a cDNA whose sequence was most similar to teleost *zic2* in the NCBI database (*Gasterosteus aculeatus*, three-spined stickleback; e-value = 0.0 and *Danio rerio*,zebrafish; e-value = 0.0). Zebrafish have been shown to possess two *zic2* homologs, *zic2a (zic2.1)* and *zic2b (zic2.2)*, due to genome duplication [4], [27]. The bluehead wrasse *zic2* cDNA shows the strongest sequence identity to the zebrafish *zic2a* gene [27]. Initial NCBI BLAST results of the *Thalassoma bifasciatum zic2* (HQ423137.1) sequence indicated 84% sequence identity to *Danio rerio zic2a* (NM131558.1) and 91% sequence identity to *Gasterosteus aculeatus zic2* (BT027912.1). Nucleotide sequence alignment of the confirmed z*ic2* partial nucleotide sequence isolated from *Thalassoma bifasciatum* (bluehead wrasse; HQ423137.1) aligned with *Gasterosteus aculeatus zic2* (three-spined stickleback; BT027912.1) is presented in supporting information (Figure S1). Comparison of amino acid sequence alignments using ClustalW2 software (European Bioinformatics Institute, Cambridge, UK) indicated 95% sequence identity between bluehead wrasse *zic2* and *Danio rerio zic2a*. However, an unrooted Bayesian phylogenetic tree of vertebrate *zic2* protein sequences does not clearly demonstrate a closer relationship of *Thalassoma zic2* (Figure S2, Text S1) to zebrafish *zic2a* than to zebrafish *zic2b*. Furthermore, BLAST searches to identify homologs of zebrafish *zic2b* were unsuccessful, so it is not entirely clear that distinct *zic2a* and *zic2b* genes are a general feature of teleost genomes. Genome sequencing efforts for a variety of teleosts include bluehead wrasses should resolve this issue relatively soon but, given these uncertainties, we take a conservative approach and refer to the transcript characterized here as *Thalassoma bifasciatum zic2*.

### *Zic2* mRNA localization and mapping

**Figure 1:**
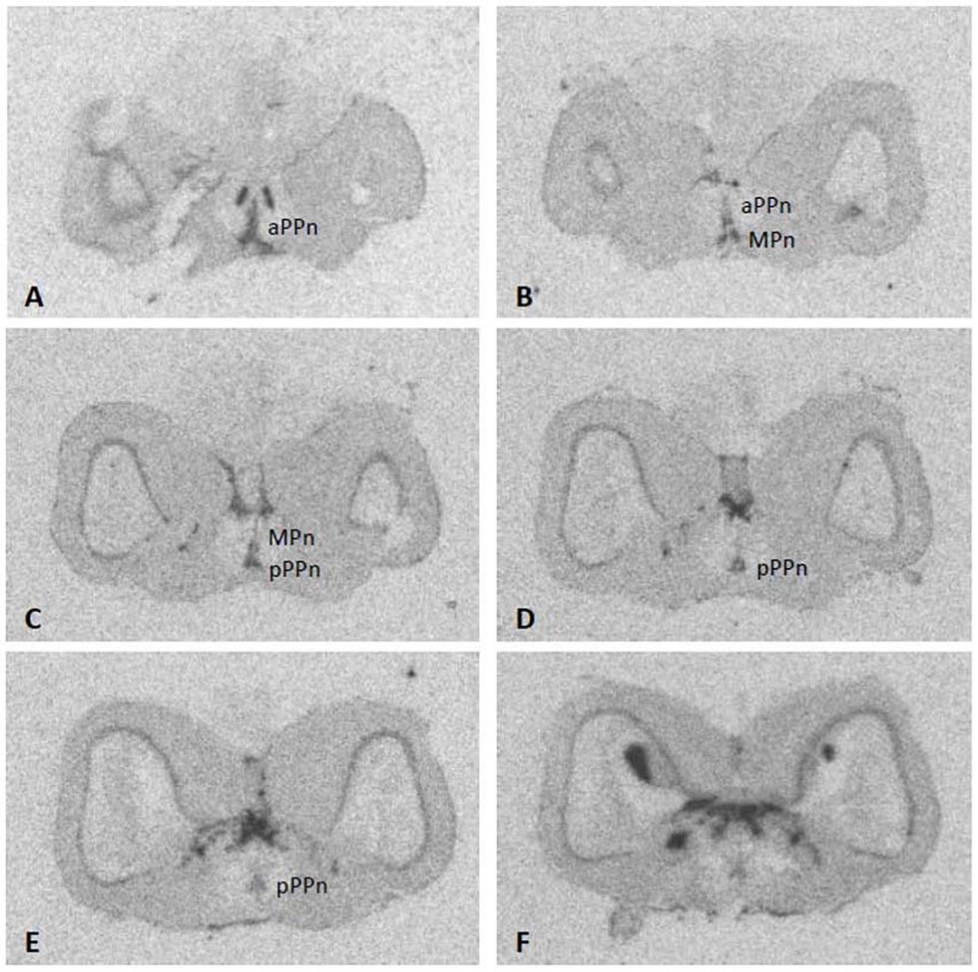
Autoradiographic images demonstrating *zic2* signal throughout the preoptic area of the hypothalamus and forebrain (A–F). Anterior parvocellular (aPPn), magnocellular preoptic nuclei (MPn), and posterior parvocellular preoptic nuclei (pPPn) of the hypothalamus.

**Figure 2:**
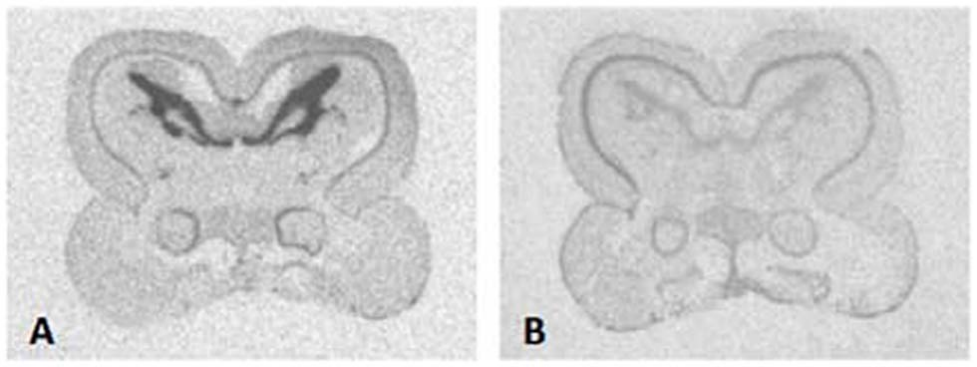
Control treatment demonstrating *zic2* antisense probe (A) compared to sense strand probe (B). Images taken on autoradiographic film.

Hybridization signal for *zic2* mRNA was observed throughout the forebrain, midbrain, and hindbrain in autoradiograms (Figure 1). Control sections hybridized with a *zic2* sense probe showed that labeling was non-specific only in the strongly Nissl-positive glomerular nucleus and optic tectum (Figure 2).

**Figure 3:**
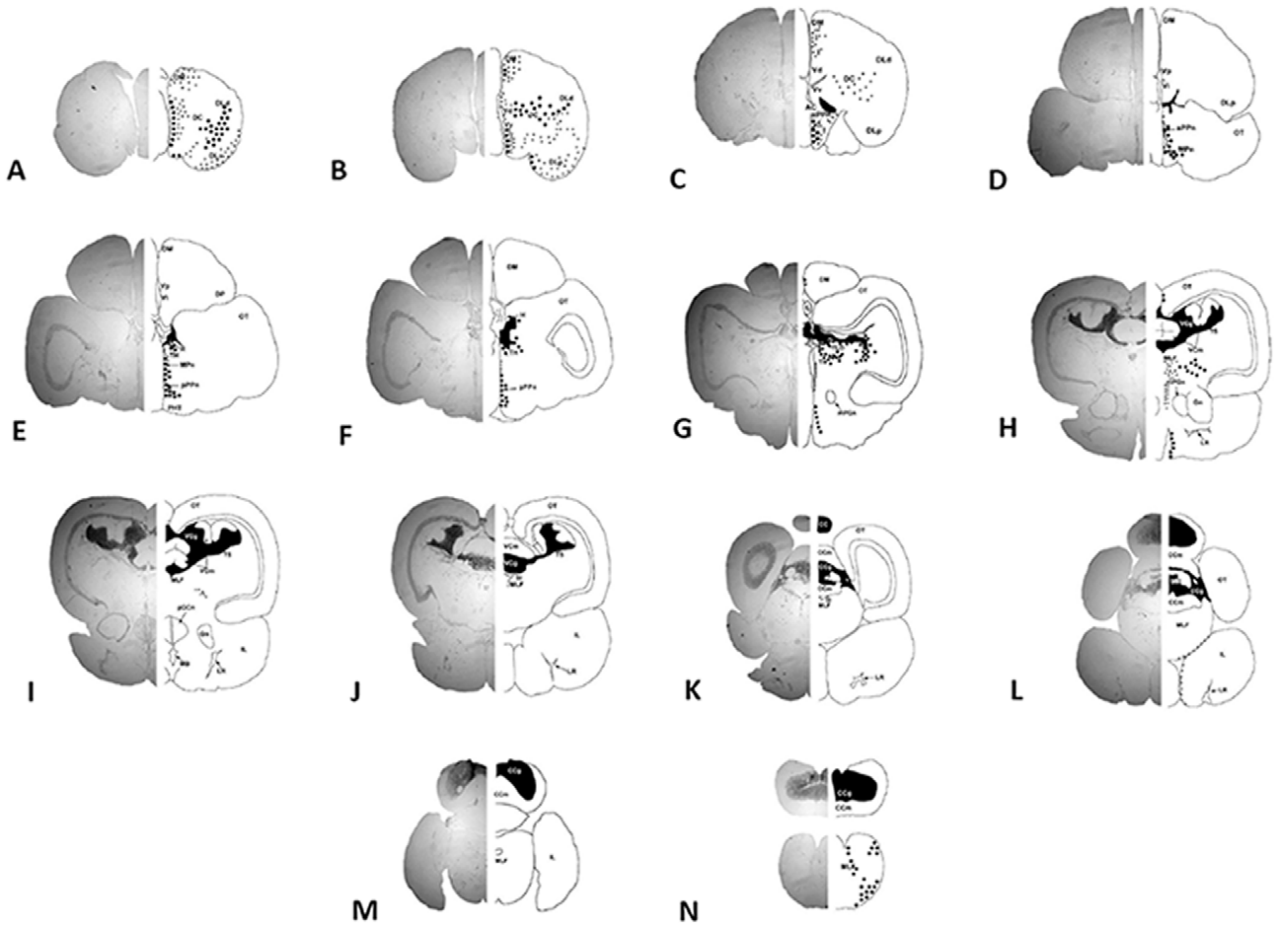
Neuroanatomical distribution of *zic2* mRNA on representative coronal sections (A–N) of the bluehead wrasse brain. Relative abundance and localization of *zic2* signal is indicated on the right side of the midline along with structure labels. Black-shaded areas indicate a solid layer of signaling, large circles indicate distinct cell body labeling, while smaller circles indicate the presence of diffuse hybridization signal not clearly restricted to distinct cells. Abbreviations in Table 1.

Hybridization signal was widespread and diffuse in the telencephalon. Signal was found in the central nucleus, medial zone, and posterior portions of the lateral zone of the telencephalic area dorsalis, the ventral and dorsal zone of the telencephalic area ventralis, and the dorsal ventral division of the lateral zone of the area dorsalis (Figure 1A; Figure 3A–C). In the telencephalon, large labeled neurons were distributed from the central nucleus of the telencephalic area dorsalis to the ventral division of the lateral zone of the telencephalic area dorsalis, as well as along the walls of the telencephalic ventricle (Figure 3A–B). Hybridization signal was visible spanning from the posterior telencephalon towards the olfactory bulb. In the preoptic area of the hypothalamus, numerous prominent neurons expressing *zic2* were observed throughout the magnocellular and anterior and posterior parvocellular preoptic nuclei (Figure 1A–D; Figure 3C–F). Just lateral to the anterior commissure and anterior preoptic area and ventral to the ventral zone of the telencephalic area ventralis, strong uninterrupted hybridization signal in the entopeduncular nucleus was mirrored in both hemispheres (Figure 1A; Figure 3C). Strong hybridization signal was observed throughout the habenula and along its boundaries extending into thalamic nuclei (Figure 1B–D; Figure 3D–F). Further into the midbrain, there was a dense layer of hybridization signal in the torus semicircularis and torus longitudinalis, as well the presence of more discrete labeling just ventral to the torus semicircularis (Figure 1E–F; Figure 3G–H). In these regions, the ventral portion of the posterior commissure exhibited large *zic2 mRNA-*labeled cells (Figure 3G–H). Medial to the preglomerular nuclei and along the midline, diffuse signal stretched into the medial longitudinal fascicle (Figure 3H). In the hindbrain, a strongly-labeled layer of signal extended throughout the granule cell layer of the valvula cerebelli and cerebellum, as well as the crista cerebellaris (Figure 3N). *Zic2* mRNA was found lining the ventral tegmental commissure and inferior lobe of the hypothalamus (Figure 3L), and signal was observed in the medial longitudinal fascicle ventral to the cerebellum (Figure 3N).

### *Zic2* mRNA abundances across sexual phenotypes

**Figure 4:**
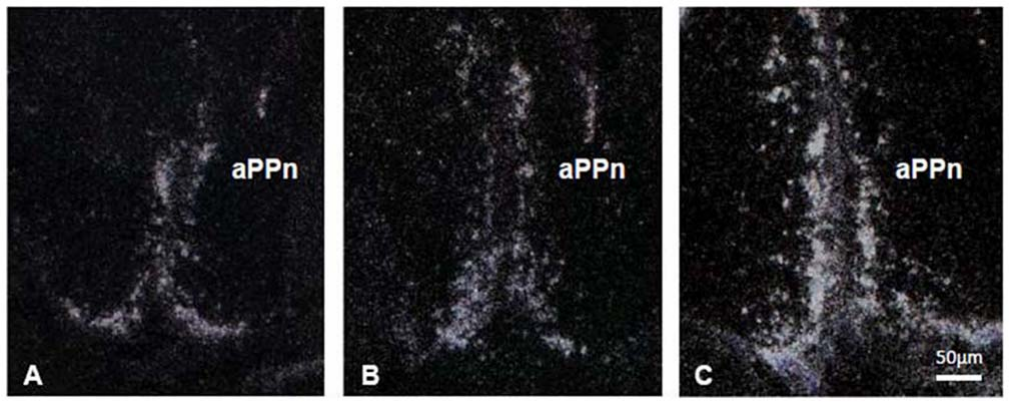
Expression of zic2 mRNA in the anterior parvocellular preoptic nucleus (aPPn) of the hypothalamus. Expression across female (A), initial phase male (B), and terminal phase male bluehead wrasse (C). Darkfield images of silver grain localization and density were taken at 100× total magnification.

**Figure 5:**
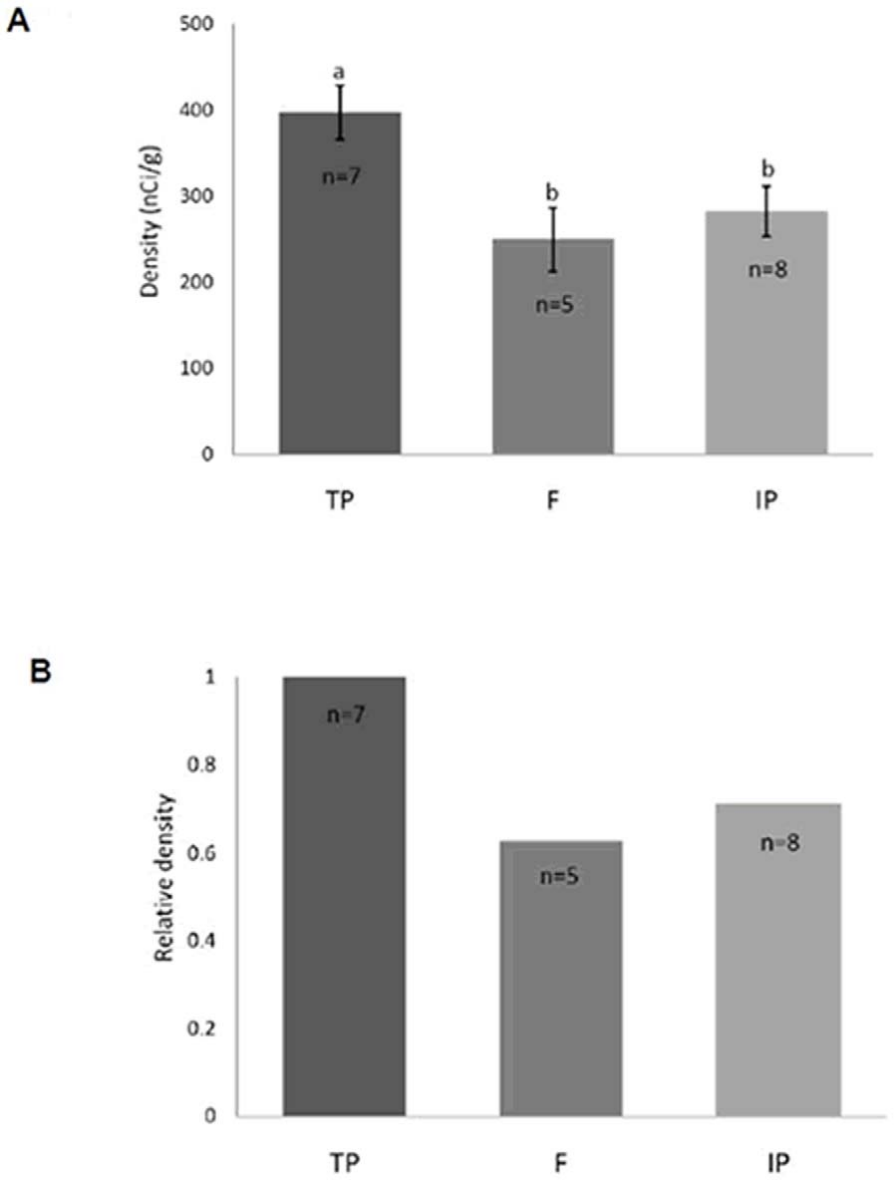
(A) Mean density of z*ic2* mRNA signal abundance and (B) relative density in the preoptic area. Comparison across phenotypes using autoradiographic quantification for terminal phase males (TP), initial phase males (IP), and females (F).

**Figure 6:**
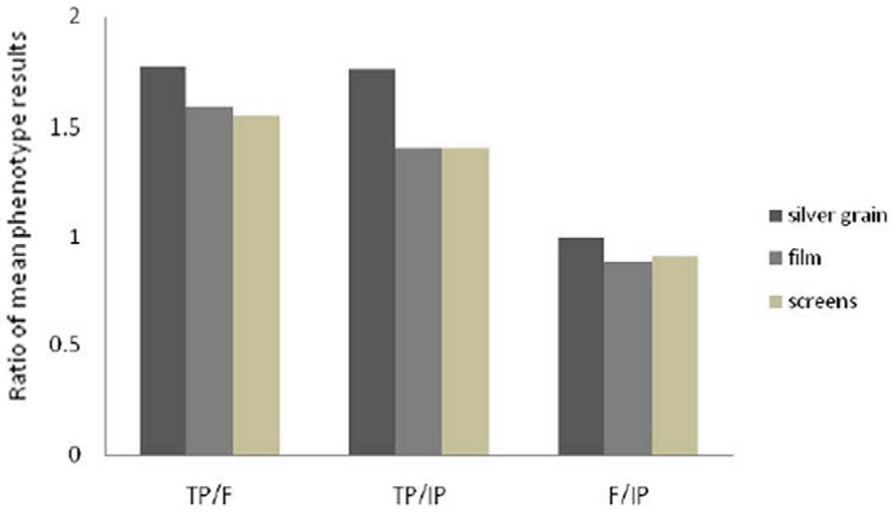
Comparison of quantification procedures for z*ic2* mRNA localized in the preoptic area of the hypothalamus. Ratios of mean signal density or mean grain totals for each phenotype was examined across techniques: silver grain counts and autoradiographic film and phosphor screen density readings. Silver grain data is based on a smaller sample size: terminal phase male (TP) (n = 6), initial phase male (IP) (n = 5), and female (F) (n = 4). Film and screen data sample size: terminal phase male (TP) (n = 7), initial phase male (IP) (n = 8), and female (F) (n = 5).

Signaling was assessed for each slide with phosphor imaging screens, autoradiographic film optical density, and silver grain quantification techniques. Clear hybridization signal was apparent in the preoptic area of the hypothalamus in all three sexual phenotypes (Figure 4). Both phosphor screen and film-based optical density (Figure 5) measures indicated higher *zic2* mRNA abundances in terminal phase males than either initial phase males or females in the preoptic area (ANOVA, autoradiographic quantification: F_2, 17_ = 5.6930, *p* = 0.0128; phosphorimaging screens: F_2, 17_ = 4.3873, *p* = 0.0291). Silver grain quantification in the preoptic area indicated the same differences between phenotypes as the film-based optical density comparisons. Grain counts in the preoptic area of terminal phase males were significantly higher than both females and initial phase males (ANOVA; F_2, 12_ = 15.2390, *p* = 0.0005; Wilcoxon signed-rank test, *p* = 0.0063). Significant correlations between i) mean absolute density values from autoradiographic film and phosphor imaging quantification (Pearson's R = 0.6976, *p* = 0.0006) and ii) ranking of values from individuals (Pearson's R = 0.7097, *p* = 0.0005) demonstrated consistency in relative values between measurement techniques. Optical density measures from film-based autoradiography also correlated well with silver grain quantification data, whether assessed by comparing optical density against silver grain measures directly or the ranking of values from individuals (absolute value: R = 0.7650, *p* = 0.0023; ranking: R = 0.7692, *p* = 0.0021). Comparison across silver grain, autoradiographic film optical densities, and phosphor screen techniques demonstrated a consistent relationship between phenotypes (ratios of means silver grains∶film∶screens; F/IP = 0.9939∶0.8827∶0.9057; TP/IP = 1.7600∶1.4066∶1.4072; TP/F = 1.7703∶1.5934∶1.5535, Figure 6). For the three techniques, phenotype was the only variable which entered into the stepwise model and explained a statistically significant part of the variation observed in quantification measures. No correlation was observed between standard length and preoptic area z*ic2* mRNA abundances (silver grain, *p* = 0.4835; film, *p* = 0.6143; screen, *p* = 0.8735). The model considered values for initial phase males and females together (silver grain, *p* = 0.9730; film, *p* = 0.4907; screen, *p* = 0.6167) and demonstrated that only the relationship between females and initial phase males to terminal phase males explained part of the variation observed (silver grain, *p* = 0.0001, R = 0.8471; film, *p* = 0.0036, R = 0.6194; screen, *p* = 0.0080, R = 0.5748). Analysis of cerebellar hybridization signal intensity across phenotypes showed no significant difference in *zic2* mRNA labeling density (ANOVA; F_2,238_ = 2.3089, *p* = 0.1016; data not shown). There were also no significant differences in *zic2* signal across phenotypes in the habenula (phosphorimaging screens; ANOVA; F_2, 17_ = 2.1764, *p* = 0.1440; data not shown).

## Discussion

*Zic* has been studied primarily in relation to development, but we found widespread expression of *zic2* mRNA in the brain of adult bluehead wrasses. Expression was high in the granule cells of the cerebellum, as in other species examined to date, but is also present in a variety of other brain areas. These areas include the thalamus, hypothalamus, habenula, torus semicircularis, torus longitudinalis, medial longitudinal fascicle, and various telencephalic regions. We also found that *zic2* mRNA was more abundant in the preoptic area of terminal phase (TP) males than in either females or the female-mimic initial phase (IP) males, but found no significant differences in *zic2* mRNA in the habenula or cerebellum across sexual phenotypes.

Consistent with the findings of Aruga et *al.*, 2002 [12], we found dense *zic2* mRNA labeling throughout the granule cell layer of the cerebellum. Additional signal was noted in the granule layer of the valvula cerebella and crista cerebellaris which, as shown in zebrafish, contain proliferating cells that give rise to cerebellar granule neurons [17]. Robust *zic2* signaling was present throughout the habenula extending into thalamic and hypothalamic nuclei. With outputs to both dopaminergic and serotonergic targets, the habenula is also an important link between the limbic and striatal forebrain in at least mammals [28], [29].

In addition to expression in sensory processing regions, *zic2* mRNA is also expressed in key integrative areas. Expression in the hypothalamus includes both the magnocellular and anterior and posterior parvocellular divisions of the preoptic area. The preoptic area is a critical region regulating reproductive behavior and function across vertebrate animals and exhibits sexual dimorphisms in a variety of species including bluehead wrasses [18], [25], [30], [31], [32]. Specifically in fishes, preoptic area lesions in male goldfish impair sexual behaviors including following, butting, and spawning activity [26] while electrical stimulation of this area can induce sexual behavior male sunfish [24]. Previously, our laboratory found differences in the expression of the behaviorally and reproductively important arginine vasoctocin (AVT) and aromatase genes in the preoptic area across sexual phenotypes [18], [22], [33]. Here, we found greater *zic2* mRNA abundances in the preoptic area of TP males than either females or IP males, suggesting *zic2* could be important in sex and role differentiation, perhaps through effects on neuroendocrine signaling or neurogenesis. The dopaminergic system has been implicated in the regulation of sexual behavior in a variety of species and two studies have examined the involvement of monoamines in sex change in the saddleback wrasse (*Thalassoma duperrey*), a congener of the bluehead wrasse [34], [35]. In the saddleback wrasse, non-specific dopaminergic receptor inhibition via haloperidol induced protogynous sex change in a socially inhibitory environment (i.e., with a larger individual present) [34]. Tyrosine hydroxlase (TH) is the rate-limiting enzyme in dopamine synthesis and TH-immunoreactive cell bodies and fibers are found throughout the preoptic area of the bluehead wrasse and other fishes [33], [36], [37]. Specifically, the co-regionalization of aromatase-ir cell bodies and fibers with TH-ir neurons has been described in the preoptic area of the bluehead wrasse [33]. *Zic2* functions as a transcriptional repressor of the human dopamine receptor D1 (DRD1, D1A) and the promoter region of this receptor is one of the few known direct targets of *zic2* [38]. Of note, work in male European starlings has found breeding context dependent densities of DRD1 in the medial preoptic area [39]. Differences in *zic2* expression in the bluehead wrasse preoptic area could potentially influence sexual behavior and function through effects on dopaminergic signaling.

Neurogenesis is also likely influenced by *zic2* in the bluehead wrasse as in other species. In the developing mammalian brain, the medial septal nucleus, the thalamic nuclei, and preoptic nucleus express *zic2* [12]. Interestingly, we observed a similar pattern of *zic2* expression in the adult bluehead wrasse brain, potentially reflecting the neural plasticity and high levels of neurogenesis that adult teleosts exhibit. Consistent with this suggestion, regions of cellular proliferation and neurogenesis in the adult zebrafish [40] are in agreement with those observed during development in mammals and *zic2* mRNA expression in the adult bluehead wrasse. Specifically, these include the telencephalon, preoptic area, habenula, thalamus, ventricular domain of the hypothalamus, torus longitudinalis, corpus cerebella, valvula cerebella, and cerebellum [40]. In the adult mouse brain, *zic2* expression overlaps with markers of neurogenesis in the dentate gyrus of the hippocampus and subventricular zone lining the lateral ventricles [17]. Consistent with Aruga and collaborators [14] and Brown and Brown [17], we found *zic2* expression in the telencephalon of adult blueheads. The lateral and posterior zones of the telencephalon in teleosts potentially represent a functional homolog of the mammalian hippocampus, and there are descriptions of cellular proliferation and neurogenesis in these regions in fishes [40]. Grober and Bass [41] found greater numbers of GnRH-immunoreactive neurons in TP males than in females or IP males in the bluehead and this is also true for vasotocin neurons [18], [22]. It is possible that neurogenesis is important for these reproductively and behaviorally-related neuroendocrine changes, although this has not been clearly established.

In summary, *zic2* is widely expressed in the adult bluehead wrasse brain, including regions that regulate sexual behavior and function. The function of *zic2* remains relatively unstudied in adult animals generally and this work is therefore novel in adult teleosts. We have also provided evidence of sexual phenotype differences in *zic2* expression in the preoptic area. Together with evidence of roles in neural differentiation and dopaminergic signaling in other species, these sexual phenotype differences in bluehead wrasses suggest a potential role in their remarkable sexual plasticity. Further research involving mechanistic approaches will be necessary to explore this possibility.

## Materials and Methods

### Study site and species

Adult bluehead wrasses (*Thalassoma bifascitum*) were collected from shallow patch reefs in the Florida Keys National Marine Sanctuary near Key Largo, Florida (reefs in the area of 25°13′W, 80°14′W) under permit during July 2008. Females, IP males, and TP males were captured using a lift net between 0900h–1300h over two consecutive days (see references 21 and 23 for descriptions of lift netting). In order to ensure accurate behavioral phenotyping, fish were observed before capture to verify spawning activity. Captured fish were sacrificed immediately (within three minutes) in a small boat at the capture site using an overdose of MS-222 (tricaine methanesulfonate, Sigma, St. Louis, MO). Before sacrifice, fish were measured (standard length; mean (range): F = 69.0 mm (62.3–75.8 mm), IP = 68.9 mm (61.4–82.9 mm), TP = 88.9 mm (84.2–94.0 mm) and sexed by visually examining the genital papillae and/or expression of sperm or eggs following manual pressure on the abdomen. Sex was confirmed by direct examination of the gonads on disssection post-sacrifice. Brain dissection and removal was complete within approximately three minutes post sacrifice. Brains were snap frozen and kept on dry ice for transport to the laboratory at NCSU, then stored at −80°C until further processing. Brains were embedded in OCT compound (Tissutek, Durham, NC), coronally cryosectioned at 20 µm, and transferred onto Superfrost slides (Fisher Scientific, St. Louis, MO). In order to allow comparison of antisense and control sense strand treatments across adjacent sections in *in situ* hybridization, consecutive sections were thaw mounted on a series of six alternating slides. All slides were stored at −80°C until used for *in situ* hybridization. All experimental methods were in compliance with the guidelines of the Institutional Animal Care and Use Committee of North Carolina State University (NCSU).

### Preparation of riboprobe template DNA

Previous microarray work in our lab generated a bluehead wrasse brain cDNA library (pBluescript vector; Stratagene, La Jolla, CA) from field-collected fish. *Zic2* cDNA was amplified from a plasmid isolated from this cDNA library PCR product size was estimated on a 1% agarose gel and this product was purified with a QIAquick PCR Purification Kit (Qiagen, Valencia, CA), and sequenced. Comparison of the 727 bp sequence using BLAST (NCBI; sequence submission HQ423137) showed greatest sequence similarity to *Gasterosteus aculeatus* (three-spined stickleback; 91%; BT027912.1) and *Danio rerio zic2a* (zebrafish; 84%; AF151535.1) and supported the identification as *zic2*. Purified PCR product was transformed into JM109 competent cells (Sigma, St. Louis, MO). Upon confirmation of the *zic2* insert, plasmid was either linearized with ECOR1 for antisense template or HINDIII for sense template. To further assess probe specificity, an alternate 315 bp antisense *zic2* template was cut from the same plasmid and used in *in situ* hybridization.

### Probe reaction and *in situ* hybridization

Riboprobe *in situ* hybridization was used to compare the relative abundance of brain *zic2* mRNA across sexual phenotypes. The protocol followed was described previously for assessing mRNA abundances in rat brain tissue [42]. The *in situ* hybridization was performed using a 727 bp ^35^S-labeled RNA probe transcribed from template *zic2* DNA. The RNA probe was synthesized with a MAXIscript In Vitro Transcription Kit (Ambion, Austin, TX) and labeled with [α-^35^S]CTP (1250 Ci/mmol, 70 mCi/ml; Perkin Elmer, Boston, MA). Antisense probe from both 727 bp and 315 bp *zic2* template was transcribed using T7 RNA polymerase with the MAXIscript kit, while sense probe was transcribed using T3 Polymerase (Promega, Madison, WI). Probe reactions were ethanol precipitated overnight and kept at −20°C for 24 hrs until addition to hybridization buffer. RNAse control slides went through an additional one hour ribonuclease A digestion step (60 µg/ml, 37°C) (Sigma, St. Louis, MO) during standard washes that followed overnight pre-hybridization. Probe was added at 190 ng/µl/kb (0.19 µg/kb) for each reaction. A probe concentration level of 170 ng/µl/kb (0.17 µg/kb) was previously shown to be saturating through quantification of *zic2* mRNA hybridization signal in the cerebellum (the region of strongest expression) across a range of probe concentrations. Following post-hybridization washes, slides were dried and placed on Kodak BioMax MR film for a 73 hour exposure at room temperature (Kodak, Rochester, NY). After film exposure, slides were placed on Kodak phosphor screens (Carestream Health, New Haven, CT) for five days to enable radioisotopic hybridization signal measurement on a Bio-Rad Molecular Imager FX scanner (Bio-Rad Laboratories, Inc., Hercules, CA). Two weeks post-processing, slides were dipped in Kodak NTB3 autoradiographic emulsion (Carestream Health, New Haven, CT), dried, and exposed in a light-sealed box at 4°C for three weeks. Exposed slides were developed for four minutes (Kodak D19; Kodak, Rochester, NY), rinsed briefly with running water, and fixed for 12 minutes (Kodak Fixer; Kodak, Rochester, NY) under safelight conditions. After rinsing for approximately 20 minutes under running water, slides were cresyl violet stained and coverslipped with Permount (Fisher Scientific, St. Louis, MO) to observe morphology. All images were taken using a Retiga 2000R, 12 Bit Color Camera (QImaging, Surrey, BC, Canada) and MCID Image Capture software (InterFocus Imaging Ltd., Linton, Cambridge, UK).

### Localization of signal

Comparison of adjacent sense strand sections to antisense sections from the same animal was utilized to correct for background and identify potential non-specific labeling (Figure 2). Control treatment with a sense-strand probe showed non-specific labeling only in the glomerular nucleus and optic tectum. The alternate, 315 bp antisense *zic2* probe (BAMH1digested clone; Promega, Madison, WI) showed identical patterns of labeling to the probe generated from the 727 bp *zic2* template, and pre-digestion with RNaseA eliminated labeling. A similar protocol was used to compare relative labeling densities from film exposure of probe dilution test slides. Cerebellar optical density readings across comparable sections from the same animal were submitted to a Wilcoxon signed-rank nonparametric test (SAS, Cary, NC) in this control. A dilution test intended to ensure use of sufficient probe to saturate endogenous *zic2* mRNA indicated comparable hybridization signal levels in the cerebellum with probe concentrations at 0.340 ng/ml/kb, 0.170 ng/ml/kb, and 0.085 ng/ml/kb (Wilcoxon signed-rank test, *p* = 0.6977).

Distribution of *zic2* mRNA was mapped in detail for a terminal phase (TP) male bluehead wrasse by comparison of antisense and sense-strand control slides from the same animal. This distribution was compared against other animals of the TP male, IP male, and female phenotypes to assess consistency of *zic2* mRNA localization (24 animals; eight of each phenotype). Neuroanatomical nomenclature is consistent with Wullimann et *al.* [43] and previous work from our laboratory [33]. Bright field and dark field images were obtained using a Leica digital microscope (Leica Microsystems, Bannockburn, IL) with a Retiga 2000R, 12 Bit Color Camera (QImaging, Surrey, BC, Canada) and MCID image analysis software (InterFocus Imaging Ltd., Linton, Cambridge, UK). Images were adjusted for light and contrast in Windows Photo Gallery (Microsoft Corporation, Redmond, Washington). ClustalW version 2.0.10 (European Bioinformatics Institute, Cambridge, UK) was utilized for aligning multiple nucleotide and amino acid sequences.

### Quantification of hybridization signal and comparisons across sexual phenotypes

Three different quantification procedures were utilized to assess precision and consistency of the results generated. These methods were quantitative regional autoradiography (optical density estimated from film), silver grain quantification, and relative hybridization signal measures from phosphor imaging screens. Quantitative regional autoradiography using MCID software (InterFocus Imaging Ltd., Linton, Cambridge, UK) was employed for a high resolution measurement of signal density (indicating relative hybridization signal) in the cerebellum and preoptic area of the hypothalamus across phenotypes. Labeling density in the cerebellum was quantified and compared across twenty-four animals: eight animals from each of the three phenotypes. Density was quantified in ten sections of the granule cell layer of the cerebellum. A rotatable ellipse (100×75 µm) drawn using MCID software was utilized for the quantification of this region. The elliptically-shaped template was designed to lie within the confines of the granule cell layer in the cerebellum, and template placement was kept consistent across all sections and animals. In the preoptic area, typically, four sections from each animal were used for quantification of optical density, but this ranged from two to six, with sample sizes for the different sexual phenotypes totaling five females, eight initial phase males, and seven terminal phase males. Sample size was lower than cerebellar quantification due to tissue quality or loss of the region of interest in some slide sets. An elliptically-shaped template (120×75 µm) was designed to tightly border visible signal in the preoptic area of the smallest animals. This procedure allowed for conservative measures to be obtained from larger animals, and central placement of the template along the vertically-oriented third ventricle in a position encompassing the entire preoptic area addressed concerns of sampling region uniformity. The area of quantification and placement of the template was kept constant across all sections and animals to ensure accuracy of quantification procedures in both the cerebellum and preoptic area. Background density measures, taken immediately adjacent to the template with the same ellipse, were subtracted from each reading, and density values were recorded for all sections exhibiting hybridization signal.

We used Quantity One 4.6.6 analysis software (Bio-Rad Laboratories, Inc., Hercules, CA) for measurement of hybridization signal from phosphor screen scans. A 203 µm^2^ circular region of interest was defined over sections showing signal in the preoptic area of the hypothalamus and habenula (following the quantification procedure described), and the hybridization signal of each target area was computed in counts/mm^2^ by Quantity One 4.6.6. Typically, three to four sections from each animal were used for this lower resolution quantification procedure. Preoptic area hybridization signal was quantified for 5 females, 8 IP males , and 7 TP males. Habenula hybridization signal was quantified for 5 females, 8 IP males, and 7 TP males. Background difference between screens was subtracted from each measurement, and slides from the different sexual phenotypes were randomly distributed across the screens to avoid any potential confounding effect of individual screens on average measured signal intensities. The density values for the phenotypes were independent from the screens (Two-way ANOVA; F_1, 14_ = 0.3479, *p* = 0.5647) and there was no statistical interaction between the screens and phenotypes (Two-way ANOVA; F_2, 14_ = 0.4223, *p* = 0.6636).

We compared hybridization signal across phenotypes at the cellular level by measuring silver grain density in the preoptic area using MCID software (InterFocus Imaging Ltd., Linton, Cambridge, UK). Typically, four or five sections from each animal were used for quantification purposes, but this number ranged from three to six, with sample sizes for the different sexual phenotypes totaling four females, five IP males, and six TP males. A rectangle (245×65 µm) drawn using MCID software was utilized for the quantification procedure. Grain counts were recorded from only one hemisphere of each animal. The template was designed to tightly border visible signal on one side of the preoptic area of the smallest animals and was placed parallel to the third ventricle in a fixed anatomical position. The area of quantification (245×65 µm) and the placement of the template, parallel to the third ventricle and centrally within the proximal preoptic area, was kept constant across all sections. Background silver grain density was measured immediately adjacent and lateral to the sampled area utilizing the identical template and subtracted from each preoptic area measurement taken.

### Statistical analysis

Measurements from successive sections in a brain area for individual animals were averaged and mean measurements were analyzed in JMP 7.0.0 (SAS Institute, Cary, NC) by Oneway ANOVA and Tukey-Kramer post hoc tests. To assess body size effects, a multivariate stepwise regression with standard length (in fishes, the measurement from the most anterior tip of the body to the midlateral posterior edge of the hypural plate) and sexual phenotype as factors were utilized. This analysis was performed separately for the film, phosphor screen, and silver grain quantification techniques.

**Table 1:** Abbreviations for neuroanatomical structures in Figure 1 and 3.

AC	anterior commissure
CC	crista cerebellaris
CCg	granule cell layer of the cerebellum
CCm	molecular cell layer of the cerebellum
DC	central nucleus of telencephalic area dorsalis
DLp	posterior part of lateral zone of telencephalic area dorsalis
DLd	dorsal division of lateral zone of telencephalic area dorsalis
DLv	ventral division of lateral zone of telencephalic area dorsalis
DM	medial zone of telencephalic area dorsalis
DP	posterior zone of telencephalic area dorsalis
gMP	gigantocellular portion of the magnocellular preoptic nucleus
Gn	glomerular nucleus
H	habenula
IL	inferior lobe of the hypothalamus
LR	lateral recess
MB	mammillary body
MLF	medial longitudinal fascicle
MPn	magnocellular preoptic nucleus
mPGn	medial preglomerular nucleus
OT	optic tectum
PC	posterior commissure
PHT	preopticohypophysial tract
PGCn	preglomerular commissural nucleus
aPPn	anterior parvocellular preoptic nucleus
pPPn	posterior parvocellular preoptic nucleus
TH	thalamus
TLO	torus longitudinalis
TS	torus semicircularis
VCg	granule cell layer of the valvula cerebelli
VCm	molecular cell layer of the valvula cerebelli
Vd	dorsal zone of telencephalic area ventralis
Vi	intermediate zone of telencephalic area ventralis
Vp	postcommissural nucleus of telencephalic area ventralis
Vv	ventral zone of telencephalic area ventralis

## Supporting Information

Figure S1 **Nucleotide sequence alignment of the confirmed z** ***ic2a*** **partial nucleotide sequence isolated from** ***Thalassoma bifasciatum*** **(bluehead wrasse; HQ423137.1) aligned with** ***Gasterosteus aculeatus zic2a*** **(three-spined stickleback; BT027912.1).** Nucleotides indicated with “*” represent identical nucleotides when our clone was aligned with stickleback *zic2a* sequences from the NCBI database.
(TIF)

Figure S2 **Unrooted Bayesian phylogenetic tree of vertebrate** ***zic2*** **sequences.** Numbers at each node indicate 50% or greater clade credibility values. * = only partial sequence available, ** no credibility value available for central node. Scale denotes 0.1 amino acid substitutions per site.
(TIF)

Text S1 **Detailed methods for Bayesian phylogenetic analysis of** ***zic2*** **sequences.**
(PDF)
