# Supplementary figures and images for: Sexual Phenotype Differences in zic2 mRNA Abundance in the Preoptic Area of a Protogynous Teleost, Thalassoma bifasciatum
Source: PLoS One. 2011 Aug 3;6(8):e23213. doi: 10.1371/journal.pone.0023213 (PMC3149650; doi:10.1371/journal.pone.0023213)

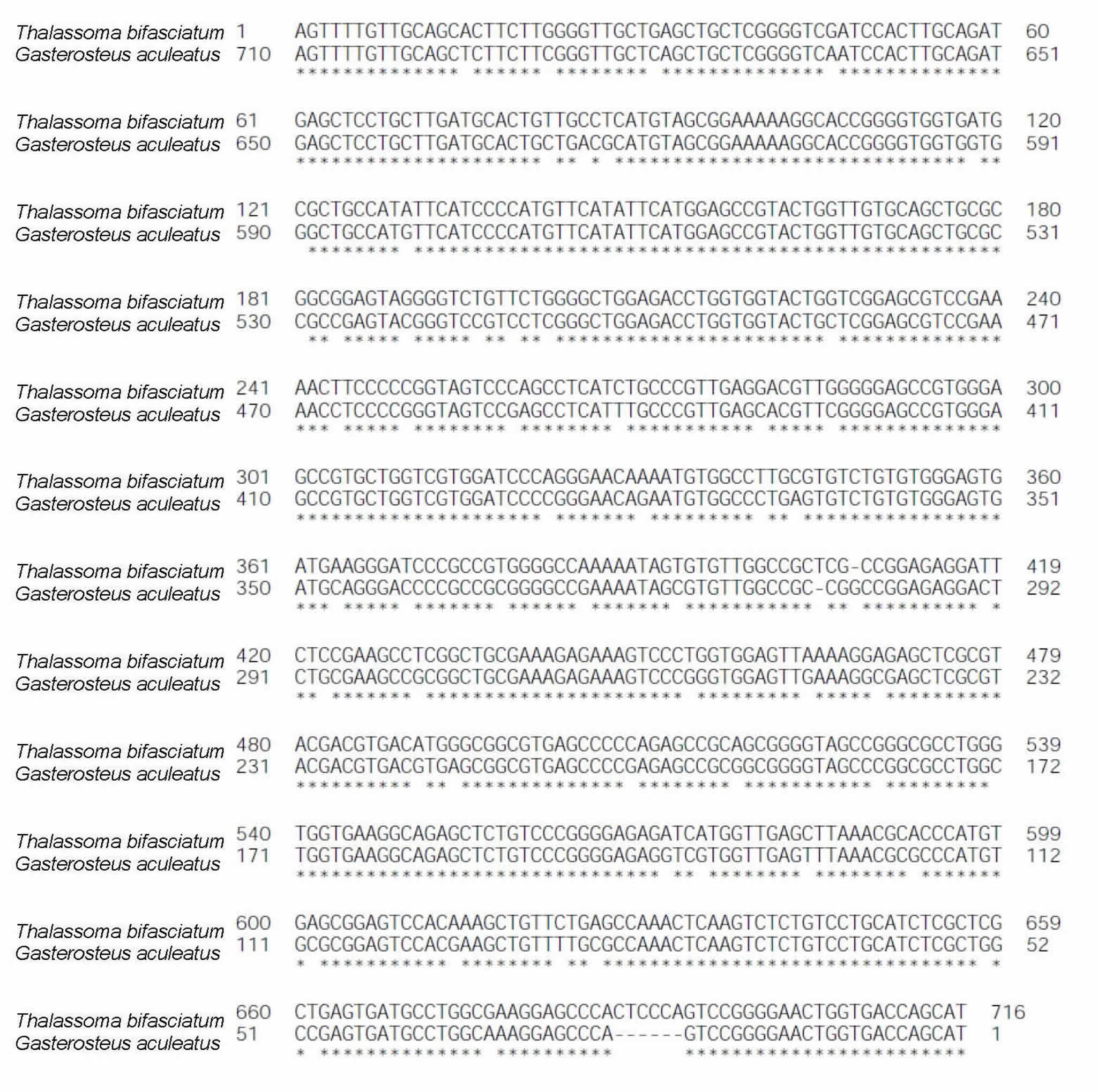

Supplement: Figure S1 — Nucleotide sequence alignment of the confirmed z ic2a partial nucleotide sequence isolated from Thalassoma bifasciatum (bluehead wrasse; HQ423137.1) aligned with Gasterosteus aculeatus zic2a (three-spined stickleback; BT027912.1). Nucleotides indicated with “*” represent identical nucleotides when our clone was aligned with stickleback zic2a sequences from the NCBI database. (TIF) [file pone.0023213.s001.tif]

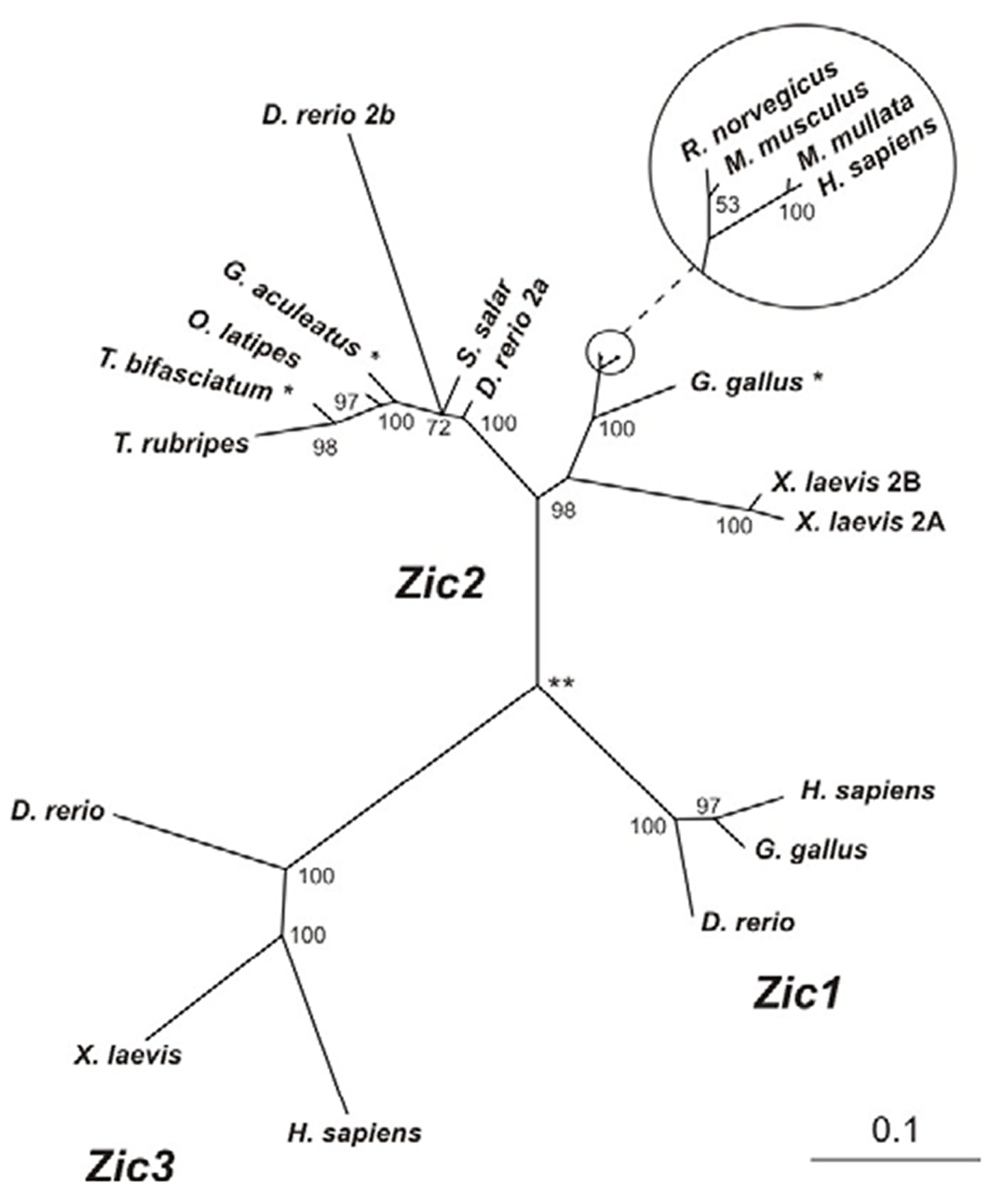

Supplement: Figure S2 — Unrooted Bayesian phylogenetic tree of vertebrate zic2 sequences. Numbers at each node indicate 50% or greater clade credibility values. * = only partial sequence available, ** no credibility value available for central node. Scale denotes 0.1 amino acid substitutions per site. (TIF) [file pone.0023213.s002.tif]
