# Supplementary material for: Sexual Phenotype Differences in zic2 mRNA Abundance in the Preoptic Area of a Protogynous Teleost, Thalassoma bifasciatum
Source: PLoS One. 2011 Aug 3;6(8):e23213. doi: 10.1371/journal.pone.0023213 (PMC3149650; doi:10.1371/journal.pone.0023213)
Supplement: Text S1 — Detailed methods for Bayesian phylogenetic analysis of zic2 sequences. (PDF) [file pone.0023213.s003.pdf]

Bayesian phylogenetic analysis of *zic2* sequences was performed online using MrBayes (v3.1.2,(Huelsenbeck and Ronquist, 2001)) using a teragrid network available through CIPRES Portal (URL: http://www.phylo.org/sub_sections/portal). The following peptide sequences were used in the analysis: *Thalassoma bifasciatum zic2* (ADZ55401.1, partial), *Salmo salar zic2* (ACN11131.1), *Mus musculus zic2* (NP_033600.3), *Macaca mulatta zic2* (XP_001093759.2), *Rattus norvegicus zic2* (NP_001101862.2), *Homo sapiens zic2* (AAG28409.1), *Xenopus laevis zic2A* (NP_001081193.1), *Xenopus laevis zic2B* (NP_001079428.1), *Danio rerio zic2a* (NP_571633.1), *Danio rerio zic2b* (NP_001001820.1), *Gallus gallus zic2* (derived from predicted genomic sequence XR_026941.1, partial, CDS position 4...953, translated in VNTI (Lu and Moriyama, 2004)), *Gasterosteus aculeatus zic2* (derived from cDNA clone BT027912.1, partial, CDS position 3..1316, translated in VNTI), *Takifugu rubripes* zics (ENSTRUG00000011863), *Oryzias latipes zic2* (ENSORLG00000011117). *Oryzias latipes* and *T. rubripes zic2* was found at http://www.ensembl.org/(genome); EMBL and Wellcome Trust Sanger Institute. For outgroup comparison, three peptide sequences for both *zic1* and *zic3* were also used: *Homo sapiens zic3* (NP_003404.1), *Danio rerio zic3* (NP_001001950.1), *Xenopus laevis zic3* (NP_001081088.1), *Gallus gallus zic1* (NP_989585.1), *Homo sapiens zic1 (*NP_003403.2), *Danio rerio zic1*(NP_571008.1). The peptide sequences were aligned using ClustalX (Thompson et al., 1997). Bayesian phylogenetic analysis was performed using the WAG amino acid substitution model and equal among-site rate variation with tree-sampling every 100th generation (burnin = 50)(Whelan and Goldman, 2001; Huelsenbeck and Ronquist, 2001). The two cold-chain runs reached 118,000 generations before terminating when the standard deviation of split-frequencies fell below 0.01. Sampled trees (2,262 from both runs) were used to create a consensus treefile with 50% or greater credibility (support for each node). The taxon names and clade support values were modified *post hoc* for illustrative purposes only using TreeView and CorelDraw (v12) (Page, 1996). No other modifications were performed.

Huelsenbeck JP, Ronquist F (2001) MRBAYES: Bayesian inference of phylogenetic trees. Bioinformatics. 17:754-755.

Lu G, Moriyama EN (2004) Vector NTI, a balanced all-in-one sequence analysis suite. Briefings in Bioinformatics. 5:378-88.

Page RDM (1996) Tree View: An application to display phylogenetic trees on personal computers. CABIOS. 12:357-358.

Thompson JD, Gibson TJ, Plewniak F, Jeanmougin F, Higgins DG (1997) The CLUSTAL_X windows interface: flexible strategies for multiple sequence alignment aided by quality analysis tools. Nucleic Acids Res. 25:4876-82.

Whelan S, Goldman N (2001) A general empirical model of protein evolution derived from multiple protein families using a maximum-likelihood approach. Mol Biol Evol. 18:691-9.
